# Valorization of pyrolysis water: a biorefinery side stream, for 1,2-propanediol production with engineered *Corynebacterium glutamicum*

**DOI:** 10.1186/s13068-017-0969-8

**Published:** 2017-11-21

**Authors:** Julian Lange, Felix Müller, Kerstin Bernecker, Nicolaus Dahmen, Ralf Takors, Bastian Blombach

**Affiliations:** 1 Institute of Biochemical Engineering, University of Stuttgart, 70569 Stuttgart, Germany; 2 Institute for Catalysis Research and Technology, Karlsruhe Institute of Technology (KIT), 76344 Eggenstein-Leopoldshafen, Germany

**Keywords:** *Corynebacterium glutamicum*, Bioeconomy, Biorefinery, Lignocellulose, Fast pyrolysis, Pyrolysis water, Metabolic engineering, 1,2-propanediol (propylene glycol), Growth-coupled biotransformation

## Abstract

**Background:**

A future bioeconomy relies on the efficient use of renewable resources for energy and material product supply. In this context, biorefineries have been developed and play a key role in converting lignocellulosic residues. Although a holistic use of the biomass feed is desired, side streams evoke in current biorefinery approaches. To ensure profitability, efficiency, and sustainability of the overall conversion process, a meaningful valorization of these materials is needed. Here, a so far unexploited side stream derived from fast pyrolysis of wheat straw—pyrolysis water—was used for production of 1,2-propanediol in microbial fermentation with engineered *Corynebacterium glutamicum*.

**Results:**

A protocol for pretreatment of pyrolysis water was established and enabled growth on its major constituents, acetate and acetol, with rates up to 0.36 ± 0.04 h^−1^. To convert acetol to 1,2-propanediol, the plasmid pJUL*gldA* expressing the glycerol dehydrogenase from *Escherichia coli* was introduced into *C. glutamicum*. 1,2-propanediol was formed in a growth-coupled biotransformation and production was further increased by construction of *C. glutamicum* Δ*pqo* Δ*aceE* Δ*ldhA* Δ*mdh* pJUL*gldA*. In a two-phase aerobic/microaerobic fed-batch process with pyrolysis water as substrate, this strain produced 18.3 ± 1.2 mM 1,2-propanediol with a yield of 0.96 ± 0.05 mol 1,2-propanediol per mol acetol and showed an overall volumetric productivity of 1.4 ± 0.1 mmol 1,2-propanediol L^−1^ h^−1^.

**Conclusions:**

This study implements microbial fermentation into a biorefinery based on pyrolytic liquefaction of lignocellulosic biomass and accesses a novel value chain by valorizing the side stream pyrolysis water for 1,2-PDO production with engineered *C. glutamicum*. The established bioprocess operated at maximal product yield and accomplished the so far highest overall volumetric productivity for microbial 1,2-PDO production with an engineered producer strain. Besides, the results highlight the potential of microbial conversion of this biorefinery side stream to other valuable products.

**Electronic supplementary material:**

The online version of this article (10.1186/s13068-017-0969-8) contains supplementary material, which is available to authorized users.

## Background

In near future, the world faces severe societal challenges due to a shortage of fossil fuels, a steadily increasing world population and the impact of the global climate change. On this account, our economy must change from being mainly non-regenerative, towards a bioeconomy generating alternative product value chains, transportation and energies that rely on renewable resources and mitigate greenhouse gas emissions. In the last decades, this caused an international stir that motivated already more than forty countries to actively follow bioeconomic strategies [1]. A common focal point is the substitution of petrochemical refineries by biorefineries, which were defined and are intended to be widely developed to market maturity through the International Energy Agency (IEA) Task 42 [2]. Numerous biorefinery approaches for the conversion of non-food second generation lignocellulosic biomasses have been developed and extensively analyzed (e.g., reviewed in [3, 4]).

Among them, fast pyrolysis has gained much attention due to the high yield of liquid bio-oil product (e.g., reviewed in [5, 6]) and constitutes a thermal liquefaction process of biomass under oxygen exclusion. Bio-oils can be utilized in different ways, from heat generation (state-of-the-art today), as refinery feed after catalytic upgrading or for synthetic fuels production as developed within the bioliq^®^ process at the KIT (Karlsruhe Institute of Technology, Germany). At this plant, fast pyrolysis is evaluated in pilot scale [7, 8]. There, wheat straw as low-quality and ash-rich bioresource is mixed with hot sand and pyrolysis occurs at 500 °C within seconds. This treatment yields products according to a mass balance: liquid [34% (w/w) bio-oil with 15% (w/w) water content, 25% (w/w) pyrolysis water (PW) with 80% (w/w) water content], solid [20% (w/w) char and ash], and pyrolysis gas [20% (w/w), non-condensable] [7]. The bio-oil contains in most advanced approaches up to 60% of the initial biomass energy [8] and comprises polar organic solvents, e.g., alcohols, acids and ketones and water insoluble contents (mostly lignin-derived oligomers, but also resins and fatty acids) [7, 9]. In the bioliq^®^ strategy, bio-oil and char are mixed to obtain a bio-slurry that is further used for centralized gasification to generate syngas for chemical synthesis [7]. Aqueous condensates, i.e., PW, generally arise during fast pyrolysis as low-value side-product, especially from conversion of ash-rich and low-quality biomass like wheat straw, due to bio-oil instabilities and phase separation [9, 10]. A direct use of aqueous condensates for gasification, however, is due to the low heating value not efficient [10]. Alternative valorization of PW was for example proposed by upgrading the biomass feed [11], mixing with bio-slurries (biosyncrude) prior to gasification [7] or even for food applications in liquid smoke flavors [9]. In this work, we conceived a biotechnological approach for the valorization of PW in a growth-coupled biotransformation to manufacture 1,2-propanediol (1,2-PDO, propylene glycol) with *Corynebacterium glutamicum*.

1,2-PDO reaches a global production capacity of around 1.4 million tons per year and is used as an eco-friendly and safe chemical in antifreeze, deicing and heat transfer fluids, but also for example in pharmaceuticals, food, cosmetics, or dyes [12]. Current commercial strategies for chemical 1,2-PDO production mostly apply natural gas conversion via propylene to 1,2-PDO [13]. Due to a future depletion of fossil resources, alternative chemical synthesis strategies were developed and primarily rely on glycerol that emerges as side stream during biodiesel production [14]. As an alternative to the chemical route, aerobic and anaerobic microbial 1,2-PDO production processes were designed (e.g., reviewed in [15, 16]). A spectrum of substrates (e.g., glucose, glycerol, and lactate) for microbial manufacturing was evaluated with natural producers (e.g., *Clostridium thermosaccharolyticum, Lactobacillus buchneri*) or engineered microbial hosts (e.g., *C. glutamicum*, *Escherichia coli*, *Saccharomyces cerevisiae*) (cf. Table 1). In the prevalent biochemical route, the glycolysis intermediate dihydroxyacetone phosphate is converted to methylglyoxal by the methylglyoxal synthase (MgsA), which is further reduced to acetol or lactaldehyde by the alcohol dehydrogenase (YqhD) and/or the glycerol dehydrogenase (GldA) and eventually converted to 1,2-PDO by the GldA or the propanediol oxidoreductase/lactaldehyde reductase (FucO). The GldA enzyme of *E. coli* converts acetol to R-1,2-PDO with the use of NADH as cofactor for reduction at 100% stereoselectivity [17].

**Table 1:** Comparison of selected microbial 1,2-PDO producers

Organism	Strain, genotype, plasmid	Substrates	Oxygenation	Titer (mM)	*Y*_P/S_ (mol mol^−1^)	Process time (h)	*Q*_P_ (mmol L^−1^ h^−1^)	References
*C. glutamicum* ATCC 13032	PDO2 (Δ*pqo* Δ*aceE* Δ*ldhA* Δ*mdh* + pJUL*gldA*)	PW (acetol), YE	Aerobic/microaerobic	18	0.96	13	1.40	This study^a^
*C. glutamicum* ATCC 13032	∆*hdpA* ∆*ldh* + pEKEx3-*mgsA*-*yqhD*-*gldA*	Glucose	Aerobic	63	0.34	51	1.24	[21]
*Clostridium thermosaccharolyticum* ATCC 31960		Glucose, YE	Anaerobic	119	0.47	28^b^	4.25	[27]
*E. coli* MG1655	∆*ackA*-*pta* ∆*ldhA* ∆*dhaK* +pTHKLcfgldA mgsAyqhD	Glycerol, tryptone, YE	Anaerobic	74	0.26	72	1.02	[28]
*E. coli* MG1655	NLD294 (∆*ldhA*) + pNEA36 [*mgs*, *gldA*, *fucO*]	Glucose, YE	Anaerobic	59	0.45	60	0.99	[29]
*Lactobacillus buchneri* (LMG 6892T)		Lactate	Anaerobic	8	0.45^c^	55	0.14	[30]
*Saccharomyces cerevisiae* YPH499	pESC-URA-*mgs* & *gldA* + pESC-LEU-*gdh* & *GUP1*	Glycerol, YE, amino acids	Aerobic	29	0.26	96	0.30	[31]

^a^Parameters calculated based on the acetol found in PW

^b^Assigned from published graphic

^c^Used the given 16.5 mM of consumed lactate as basis for calculation

*Corynebacterium glutamicum* is a Gram-positive, facultatively anaerobic and generally recognized as safe organism (GRAS-status) that belongs to the class of *Actinobacteria*, and it is widely used for the industrial manufacturing of amino acids, but has also been deployed for the production of organic acids, alcohols, and specialty chemicals [18, 19]. Strains for 1,2-PDO production from glucose have also been developed more recently [20, 21]. The bacterium is highly accessible to genetic manipulation since its genome is known [22] and technologies to globally describe the cells metabolome, transcriptome, proteome, and fluxome are at hand [23]. Furthermore, *C. glutamicum* is robust, exhibits high tolerance for lignocellulose-derived inhibitors [24], and has, consequently, been considered for biorefinery applications, e.g., for organic acid or alcohol production (reviewed in [25]).

In general, biotechnological utilization of pyrolysis fractions has been proposed in the literature and is summarized in a recent review [26]. Especially, the bio-oils that represent the value product of fast pyrolysis of lignocellulosic residues and comprise sugar monomers are in focus of most investigations. With this study, we intend to broaden this perspective towards side streams such as PW, which are of low value for alternative utilization such as chemical synthesis. We exploit the biorefinery side stream PW for growth and 1,2-PDO production with wild-type and engineered *C. glutamicum* strains, respectively. Ultimately, we conceived a two-phase aerobic/microaerobic production bioprocess to improve the overall 1,2-PDO production on PW.

## Methods

### Bacterial strains, plasmids, and oligonucleotides

All utilized bacterial strains, plasmids, and oligonucleotides and their relevant characteristics or sequences and sources or purposes are listed in Table 2.

**Table 2:** List of bacterial strains, plasmids, and oligonucleotides

Strain, plasmid, or oligonucleotide	Relevant characteristics or sequence	Source, reference, or purpose
Strains		
*Escherichia coli* DH5α	F^−^ Φ80*lacZ*ΔM15 Δ(*lacZYA*-*argF*) U169 *endA1 recA1* *hsdR17* (rk^−^, mk^+^) *supE44 thi*-*1 gyrA96 relA1 phoA*	[32]
*Corynebacterium glutamicum* ATCC 13032	Wild type	American-type culture collection
*C. glutamicum* Δ*pqo* Δ*aceE* Δ*ldhA* Δ*mdh*	*C. glutamicum* carrying genetic deletions of the pyruvate:quinone oxidoreductase (*pqo*), the E1 subunit of the pyruvate dehydrogenase complex (*aceE*), the lactate dehydrogenase (*ldh*), and the malate dehydrogenase (*mdh*)	[33]
PDO1	*C. glutamicum* + pJUL*gldA*	This study
PDO2	*C. glutamicum* Δ*pqo* Δ*aceE* Δ*ldhA* Δ*mdh* + pJUL*gldA*	This study
Plasmids		
pJC4		[34]
pJUL*gldA*	pJC4::(P_*tuf*_–*gldA*–T_*rrnB*_) plasmid expressing the *E. coli* glycerol dehydrogenase (*gldA*) under control of the constitutive *C. glutamicum* EF-TU promoter (P_*tuf*_) and closed by the *E. coli rrnB* terminator (T_*rrnB*_)	This study
Oligonucleotides	5′ → 3′	
P1	GACGCCGCAGGG **TCTAGA**CCACAGGGTAGCTGGTAGTTTG	Fw primer P_*tuf*_ (pJC4, ***Xba*** **I**)
P2	CATGGTATGTCCTCCTGGACTTC	Rv primer P_*tuf*_
gldA1	GAAGTCCAGGAGGACATACCATGGACCGCATTATTCAATC	Fw primer *gldA* (P_tuf_)
gldA2	CTTCTCTCATCCGCCAAAACAGCAGGCAATTTTGCGTTC	Rv primer *gldA* (T_*rrnB*_)
T1	CTGTTTTGGCGGATGAGAGAAG	Fw primer T_*rrnB*_
T2	GATATCCATCACACTG **GCGGCCGC**AGGAGAGCGTTCACCGACAAAC	Rv primer T_*rrnB*_ (pJC4, ***Not*** **I**)
seq 1	GATCGACGGTACGCAAC	Fw sequencing primer pJUL*gldA*
seq 2	GGGTGGTAAAGGATGTCG	Rv sequencing primer pJUL*gldA*
seq 3	GCAACCTGGTTTGAAGC	Fw sequencing primer pJUL*gldA*
seq 4	GTGTTCGCTTCAATCACG	Rv sequencing primer pJUL*gldA*

For oligonucleotides, the prerequisite homologous region for Gibson assembly (underlined) and restriction sites (bold) are given and refer to the respective features or enzymes named in parenthesis

### Plasmid and strain construction

Standard molecular biology methods (e.g., agarose gel electrophoresis, restriction, PCR) were conducted according to Sambrook et al. [35]. Plasmids were isolated and PCR fragments purified with E.Z.N.A.^®^ Plasmid Mini Kit I (Omega Bio-tek, Inc., Norcross, USA) and NucleoSpin^®^ Gel and PCR Clean-up (Macherey–Nagel GmbH & Co. KG, Düren, Germany), respectively, after the manufacturer’s instructions. Using the DNeasy Blood & Tissue Kit (QIAGEN, Hilden, Germany), chromosomal DNA was extracted following the supplied protocol. Electrocompetent cells were prepared as described previously for *E. coli* [36] and *C. glutamicum* [37]. Transformation of strains with plasmids was performed for *E. coli* according to Dower et al. [36] and *C. glutamicum* including a heat shock prior to regeneration for 6 min at 46 °C [38]. Oligonucleotides were manufactured by the biomers.net GmbH (Ulm, Germany).

The plasmid pJUL*gldA*, which constitutively expresses the *E. coli* glycerol dehydrogenase gene *gldA* (Ordered Locus Names: b3945, JW5556; Gene Accession Number: ECK3937, [39]), was constructed based on the shuttle vector pJC4 [34]. The plasmid pJC4 originates in pZ1 [40], which was cloned by a fusion of the native *C. glutamicum* plasmid pHM1519 [41] and the *E.* *coli* vector pACYC177 [42, 43]. The plasmid pJC4 was linearized with *Xba*I and *Not*I and subjected to alkaline phosphatase treatment (Thermo Fisher Scientific Inc., Waltham, USA). Then, overlapping fragments containing the constitutive promoter P_*tuf*_ of the *C. glutamicum* elongation factor EF-TU (cg0587), the *gldA* gene of *E. coli*, and the strong terminator T_*rrnB*_ of the *E. coli rrnB* operon [44, 45] were amplified from the particular chromosomal DNA via PCR (Phusion Hot Start II HF DNA Polymerase, Thermo Fisher Scientific Inc., Waltham, USA; Biometra TAdvanced thermocycler, Analytik Jena, Jena, Germany) with the primer pairs P1/P2, gldA1/gldA2, and T1/T2, respectively. During amplification, the native GTG start codon of the *C. glutamicum* EF-TU was exchanged by the original ATG codon of the *E. coli gldA* gene. Isothermal plasmid assembly was achieved according to Gibson et al. [46]. The reaction batch was introduced into *E. coli* via electroporation, and the transformed cells were plated on 2× YT agar plates containing 50 µg kanamycin mL^−1^. The plasmid was verified by sequencing with the primers seq1, seq2, seq3, and seq4 (GATC Biotech AG, Konstanz, Germany) yielding pJUL*gldA* (8380 bps).

For construction of *C. glutamicum* PDO1 and PDO2, the plasmid pJUL*gldA* was isolated, transferred into *C. glutamicum* wild-type and Δ*pqo* Δ*aceE* Δ*ldhA* Δ*mdh*, respectively, and selected on BHI agar plates containing 50 µg kanamycin mL^−1^ (Bacto™ brain heart infusion, Becton, Dickinson and Company, New Jersey, USA) and 91 g sorbitol L^−1^.

### Pyrolysis water and its pretreatment

The pyrolysis water (PW) used in this study arose from fast pyrolysis of wheat straw in the bioliq^®^ plant at the KIT [8]. Initially, PW was delivered with a water content of approximately 80% (w/w) at pH ~ 2.5 and comprised water soluble organic compounds, hydrophobic entities in form of a micro-emulsion and minor suspended matter [5]. To maintain integrity, the PW was stored at − 21 °C until further use. In order to transform this liquid into a fermentable substrate, a pretreatment protocol using 100 mL PW was established. First, the pH was adjusted to 6.5 with 10 M potassium hydroxide and phase separation accelerated by subsequent centrifugation at 4500 rcf for 30 min (Centrifuge 5804 R, Rotor: A-4-44, Eppendorf AG, Hamburg, Germany). The hydrophobic upper phase was then removed with a syringe. Remnants thereof and the minor char pellet were afterwards separated from the aqueous phase by paper filtration enriched with hydrophobic interaction surface (plastic shred). This procedure yielded a clarified PW. For removal of volatile growth inhibitors such as methanol, a manually controlled heat treatment was conducted in open beakers at 80 °C on a heating plate under constant stirring. To investigate the effect of the heat load on PW composition, different incubation times of 0.5, 1, and 1.5 h were compared. The respective volume losses were considered and relative volumes of PW referred to the 0.5 h treatment [indicated as rel. % (v/v)] were applied for shaking flask experiments. Before supplementation in microbial cultivation, the PW was eventually sterile filtered (Rotilabo^®^-syringe filter, CME 0.22 μm, Carl Roth GmbH + Co. KG, Karlsruhe, Germany) and stored at − 21 °C.

### Culture media and conditions

*Escherichia coli* and *C. glutamicum* were cultivated in 2× yeast extract tryptone (YT) complex medium [35]. For growth on semi-solid media, 18 g agar L^−1^ was added. *C. glutamicum* was cultivated in modified CGXII minimal medium based on the literature [47, 48] and contained per liter 5 g (NH_4_)_2_SO_4_, 5 g urea, 21 g 3-(N-morpholino) propane sulphonic acid (MOPS), 1 g KH_2_PO_4_, 1 g K_2_HPO_4_, 0.25 g MgSO_4_·7H_2_O, 10 mg CaCl_2_, 10 mg MnSO_4_·H_2_O, 16.4 mg FeSO_4_·7H_2_O, 1 mg ZnSO_4_·7H_2_O, 0.2 mg CuSO_4_·5H_2_O, 0.02 mg NiCl_2_·6H_2_O, and 0.2 mg biotin. In CGXII* medium prepared for shaking flasks, urea was omitted and the initial pH reduced to pH 6.5. For bioreactor cultivations, the medium CGXII** (pH 7.4) did not contain urea and additionally lacked the MOPS buffer.

Further supplementation was given from sterile filtered, aqueous, and concentrated stock solutions as described in the respective experiment: 50 µg kanamycin mL^−1^ (1000×), 1 mM reduced glutathione (100×; GSH, γ-l-glutamyl-l-cysteinyl-glycine, Sigma-Aldrich Chemie GmbH, Munich, Germany), 5 g YE L^−1^ (40×; BBL™ Yeast Extract, BD, New Jersey, USA), and 5 g acetol L^−1^ (10×; hydroxyacetone, Alfa Aesar, Karlsruhe, Germany). 3.5% (v/v) clarified or 0.5 h heat treated (HT) PW was used for cultivations. For 1 and 1.5 h HT PW, relative amounts to the 0.5 h HT PW [rel. 3.5% (v/v)] were applied considering the volume loss.

*Escherichia coli* strains were cultivated in 5 mL 2× YT medium at 37 °C on a rotary shaker at 120 rpm in 15-mL test tubes. All *C. glutamicum* strains were incubated at 30 °C as 50 mL cultures in 500-mL baffled Erlenmeyer flasks. To prepare the preculture, the respective *C. glutamicum* strain was streaked on a 2× YT agar plate. After 2–3 days incubation, a 15-mL test tube containing 5 mL 2× YT medium was inoculated, cultivated on a rotary shaker at 120 rpm for 6–8 h and used to inoculate a 50 mL 2× YT overnight culture. For inoculation of the main culture in 50 mL CGXII* minimal medium to a starting biomass concentration of about 0.5 g L^−1^, an appropriate aliquot of the overnight culture was harvested by centrifugation at 4500 rcf for 10 min, resuspended in 0.9% (w/v) NaCl and added aseptically.

Bioreactor cultivations were performed in 200 mL CGXII** medium initially supplemented with rel. 3.5% (v/v) 1 h HT PW and 5 g YE L^−1^ in a parallel triple glass bioreactor system (HWS Labortechnik, Mainz, Germany) with 250 mL working volume at ambient pressure. The dissolved oxygen concentration (DO) and the pH were monitored via standard probes (Mettler-Toledo GmbH, Gießen, Germany). During cultivation, the pH was controlled at setpoint 7.4 by addition of 25% ammonium hydroxide solution and 10% ortho-phosphoric acid. To prevent excessive foaming, Struktol™ J 647 (Schill + Seilacher, Hamburg, Germany) was added manually on demand.

For aerobic intermittent fed-batch cultivations, the DO level was maintained above 35% by an increase of gassing and agitation, starting with 0.1 vvm and 100 rpm, respectively. Feeding of PW and YE was performed immediately with identical amounts as initially applied in the batch phase at an increasing DO signal, which marks the entire consumption of acetate.

In the two-phase aerobic/microaerobic intermittent fed-batch fermentation, a constant stirring of 100 rpm was applied throughout the process. In the first aerobic phase, the aeration rate was set to 0.1 vvm. After 5 h of cultivation, identical amounts of PW and YE compared to the aerobic batch phase were introduced and aeration was reduced to 0.025 vvm. The feed pulse was chosen before acetate depletion to prevent product consumption.

### Analytical methods

Biomass formation was followed by photometric scatter analysis of a biosuspension sample at 600 nm (OD_600_). A correlation to the cell dry weight (CDW [g L^−1^] = *α*·OD_600_) was determined by analyzing the dry mass in representative wild-type cultivations (CGXII** + 60 g L^−1^ glucose) using an 8-point calibration over a wide range of growth rates between 0.08 and 0.46 h^−1^. The correlation factors α were 0.22 g L^−1^ (used for shaking flask experiments; Ultrospec 10 Cell Density Meter, GE Healthcare Europe GmbH, Freiburg, Germany) and 0.30 g L^−1^ (used for bioreactor cultivations; DR 2800 Spectrophotometer, Hach Lange GmbH, Düsseldorf, Germany).

To analyze metabolite concentrations in the culture supernatant, 1 mL of the biosuspension was centrifuged at ≥ 11,000 rcf for ≥ 1 min. Concentrations of acetate, acetol, and 1,2-propanediol were determined via HPLC according to Buchholz et al. [49] using an Agilent 1200 series apparatus (Agilent Technologies, Santa Clara, USA) equipped with a Rezex™ ROA-Organic Acid H^+^ (8%) LC column (300 × 7.8 mm, 8 µm) protected by a Carbo-H^+^ SecurityGuard™ column (4 × 3 mm) that were both purchased from Phenomenex Inc. (Aschaffenburg, Germany). Phosphate precipitation was conducted as follows: 45 µL 4 M NH_3_ and 100 µL 1.2 M MgSO_4_ were added to 1000 µL culture supernatant. After 5 min of incubation, the sample was centrifuged for 5 min at 18,000 rcf and RT. 500 µL supernatant was then transferred to 500 µL 0.1 M H_2_SO_4_. After mixing and 15 min of incubation at RT, the samples were eventually centrifuged for 15 min at 18,000 rcf at RT. Isocratic chromatography was realized with 5 mM H_2_SO_4_ as mobile phase and 0.4 mL min^−1^ flow rate for 45 min at 50 °C column temperature for acetate or 20 °C for acetol and 1,2-propanediol quantification. The injection sample volume was 10 µL. Detection was achieved by an Agilent 1200 series refractive index detector at 32 °C. Peaks were quantified via an 8-level standard calibration for each analyte as external reference and implementing l-rhamnose as internal standard.

The total organic carbon (TOC) content of crude and pretreated PW was measured with a total carbon analyzer (Multi N/C 2100s, Analytik Jena, Jena, Germany) according to literature [50] and is given in g carbon L^−1^ (C-g L^−1^).

Growth rates were calculated within the exponential growth phase by linear regression in semi-logarithmic plots of the OD_600_ above the cultivation time and using a coefficient of determination (*R*-squared) value maximization strategy. Unless stated differently, the yields were calculated by linear regression of the biomass versus acetate or the 1,2-PDO versus acetol concentrations to derive the apparent biomass yield (*Y*_X/S_*) in g CDW per g acetate or the product yield (*Y*_P/S_) in mol 1,2-PDO per mol acetol, respectively. The *Y*_X/S_* based on acetate is not a true value for cultivations applying PW and YE, because cell dry mass is formed from these resources simultaneously. Biomass-specific substrate uptake rates (*q*_S_) or biomass-specific product formation rates (*q*_P_) in mmol per g CDW per h were calculated differentially for every hour of the process by using the average biomass within the time frame and the net substrate or product concentration change, respectively. Maximum rates of the overall experiment are given for comparison (*q*_S_^max^, *q*_acetate_^max^, *q*_acetol_^max^, *q*_1,2-PDO_^max^). For all calculations and graphs, errors are given by the standard deviation (SD).

## Results and discussion

### Pyrolysis water pretreatment

In the bioliq^®^ process, pyrolysis water (PW) is formed as micro-emulsion of dispersed hydrophobic phase remnants [8]. The aqueous fraction is a complex solution of carbohydrates and comprises the two major entities acetate (0.93 ± 0.02 M, 21% of the TOC) and acetol (0.50 ± 0.02 M, 17% of the TOC) (cf. Additional file 1: Table S1). To obtain a fermentable substrate, a pretreatment procedure was established separating residual oils and solids from the aqueous phase.

**Fig. 1:**
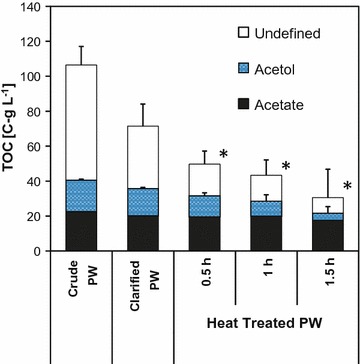
Total organic carbon (TOC) content of pyrolysis water (PW) as g carbon L^−1^ (C-g L^−1^) in the course of respective pretreatment procedures (from left to right): crude PW; clarified PW (adjustment from pH ~ 2.5 to 6.5, solid and hydrophobic phase removal); heat treated PW at 80 °C for 0.5, 1, and 1.5 h in open vessels. To allow comparability between the applied procedures, volume losses during heat treatment were considered and the concentrations of the indicated samples (*) were recalculated with respect to the initial volume (cf. Additional file 1: Figure S3A). Error bars show SD of ≥ 4 independent treatments and measurements

First, we recorded a titration curve of the PW (cf. Additional file 1: Figure S1) and observed the highest buffer capacity close to the pK_a_ of acetic acid. This indicates that acetate predominantly accounts for the low initial pH of about 2.5 of crude PW and represents the major buffering entity. Accordingly, it was previously reported for bio-oils derived from fast pyrolysis that volatile acids are responsible for 60–70% of the total acidity [51]. Adjustment of the pH to 6.5 with 10 M potassium hydroxide destabilized the micro-emulsion (cf. Additional file 1: Figure S2) due to dilution effects and polarity changes of surfactants solubilizing the micro-emulsion [5]. Subsequent centrifugation and filtration eliminated the remnant hydrophobic oil and solid char (cf. Additional file 1: Figure S2) provoking a 33 ± 6% decrease of the TOC in the clarified PW. The acetate and acetol concentrations were slightly reduced by 11 and 14%, respectively (cf. Fig. 1 and Additional file 1: Table S1). Evaporation of volatile substances (e.g., methanol) was achieved by heat treatment at 80 °C in open jars. An increase of the heat exposure time (0.5, 1, 1.5 h) led to a relative loss of the total organic carbon content (53.2 ± 8.0, 59.2 ± 11.9, 71.3 ± 37.9%), acetate (13.3 ± 2.0, 11.7 ± 2.2, 21.6 ± 2.5%), and acetol (32.5 ± 4.4%, 52.2 ± 22.5, 77.7 ± 73.9%) compared to crude PW, respectively (cf. Fig. 1, Additional file 1: Figure S3, Table S1). A drastic increase in viscosity was observed in the 1.5 h heat treated (HT) PW sample and thus restricted further application. Since the bio-oil and aqueous products of fast pyrolysis processes are thermochemically instable, the described change in color and viscosity upon heat exposure can presumably be explained by polymerization, condensation, etherification, and esterification reactions, which may also account for the considerate loss of acetol [8, 52, 53]. These reactions can furthermore be enhanced catalytically in the presence of potassium ions [5]. Previously, it was shown that the volatile acid content does not change even in long-term (24-h) heat treatment at 80 °C [53], which is in accordance with the minor loss of acetate found here.

In the current approach, the condensate after fast pyrolysis in the bioliq^®^ plant is recovered with a high organic content (condensation step 1, 80–90 °C) giving the bio-oil. Subsequent cooling to ambient temperatures (condensation step 2) finally yields the PW [7, 8]. However, the modularized design of the plant allows flexibility in the condensation cascade and gives the opportunity to generate a directly fermentable pyrolysis water fraction in future circumventing the described pretreatment.

### Pyrolysis water as substrate for growth of *C. glutamicum*

**Fig. 2:**
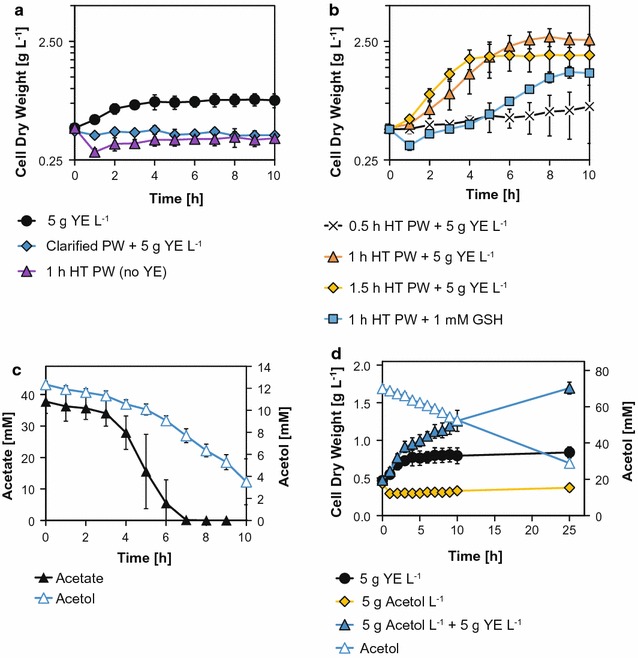
Shaking flask cultivations of *C. glutamicum* wild-type under various growth conditions. **a** Reference cultivations with supplementation of 5 g YE L^−1^ as sole carbon source (circles) as well as experiments with 3.5% (v/v) and rel. 3.5% (v/v) PW that was clarified (diamonds) or with 1 h heat treated (HT) PW without YE (triangles). **b** Growth on differently HT PW [0.5 h (crosses), 1 h (triangles), and 1.5 h (diamonds)] as well as 1 h HT PW with 1 mM reduced glutathione (GSH). **c** Course of acetate and acetol concentrations during growth on 1 h HT PW. **d** Investigation of the use of acetol as carbon source. Sole adding of acetol (diamonds) and YE (circles) is shown. The acetol consumption (open triangles) refers to the experiment of acetol and YE supplementation (triangles). Error bars represent the SD of ≥ 3 independent cultivations and measurements

We assessed the suitability of *C. glutamicum* to grow on (pretreated) PW. Direct supplementation of PW to the minimal medium lacks the possibility to investigate the bacterial growth via turbidity analyses and was therefore not further pursued. Sole addition of clarified (data not shown) as well as HT PW to the medium yielded no growth of *C. glutamicum* (for 1 h HT PW cf. Fig. 2a). For this reason, further cultivations were provided with 5 g YE L^−1^ as supplement. Still, clarification of PW was not sufficient to promote growth (cf. Fig. 2a). Cell proliferation was sustained, however, in medium containing 3.5% (v/v) and rel. 3.5% (v/v) HT PW (cf. Fig. 2b). An increase of the exposure time at 80 °C resulted in stepwise elevated growth rates (*µ*) of 0.03 ± 0.05, 0.31 ± 0.06, and 0.36 ± 0.04 h^−1^ for 0.5, 1, and 1.5 h heat treatment, respectively. The growth rates using the latter two substrates are in the range of 0.28 h^−1^ that was previously obtained in minimal medium and acetic acid as sole carbon source [54]. The highest net generated biomass of 2.39 ± 0.37 g L^−1^ accumulated by utilization of 1 h HT PW (reference cultivation on 5 g YE L^−1^ yielded 0.39 ± 0.05 g L^−1^, cf. Fig. 2 a, b). The apparent biomass/acetate yield (*Y*_X/S_*) for the 1 and 1.5 h treatment was 0.83 ± 0.05 and 0.86 ± 0.08 g CDW per g acetate (cf. Table 3), which are significantly higher to described values of about 0.23 g CDW per g acetate [54]. This indicates a biomass formation from other carbon sources, namely YE, acetol, and others available in PW. The maximum biomass-specific substrate uptake rates (*q*_S_^max^) using 1 h HT PW were 10.04 ± 1.25 mmol acetate per g CDW per h and 1.27 ± 0.28 mmol acetol per g CDW per h (cf. Table 3). In comparison, Wendisch et al. showed *q*_acetate_ values of 16.2 mmol acetate per g CDW per h in minimal medium with acetate as sole carbon source [54]. In the applied shaking flask cultivations with PW (clarified and 1 or 1.5 h heat treatment) supplemented with YE, *C. glutamicum* consumed acetate and acetol in parallel (cf. Fig. 2c, Additional file 1: Figure S4A). To investigate whether *C. glutamicum* is able to use acetol as sole carbon and energy source, we performed cultivations in CGXII* medium with 5 g acetol L^−1^ (cf. Fig. 2d). Notably, growth was only manifested upon an additional supply of 5 g YE L^−1^. In these experiments, a clear biphasic growth behavior was observed and a net biomass of 1.25 ± 0.10 g CDW L^−1^ was generated. A fast primary growth phase with a *µ* of 0.23 ± 0.02 h^−1^ was followed by a second phase at 0.03 ± 0.00 h^−1^ (cf. Fig. 2d). The end of the first phase corresponds to a cessation of cell division in the reference cultivation conducted solely on 5 g YE L^−1^, where 0.39 ± 0.01 g CDW L^−1^ were formed (*µ* = 0.16 ± 0.03 h^−1^). The YE supply cannot explain the overall elevated net generated biomass but provides components that promote the metabolization of acetol. The maximum biomass-specific acetol uptake rate (*q*_acetol_^max^) of 3.6 ± 0.7 mmol acetol per g CDW per h was about threefold higher compared to the cultivations using PW as substrate (cf. Table 3). Acetol metabolization may involve previously described reversible reactions within the methylglyoxal metabolism channeling carbon into glycolysis to the level of pyruvate [55].

**Table 3:** Parameters during shaking flask cultivations of *C. glutamicum* wild-type using pyrolysis water as substrate

		1 h HT PW + YE	1.5 h HT PW + YE	1 h HT PW + GSH	Acetol + YE
*µ*	(h^−1^)	0.31 ± 0.06	0.36 ± 0.04	0.18 ± 0.02	0.03 ± 0.01^a^
*q*_S_^max^	(mmol_acetate_ g_CDW_^−1^ h^−1^)	10.0 ± 1.2	11.1 ± 2.2	9.4 ± 0.8	–
	(mmol_acetol_ g_CDW_^−1^ h^−1^)	1.3 ± 0.3	1.0 ± 0.1	1.1 ± 0.2	3.6 ± 0.7
*Y*_X/S_*	(g_CDW_ g_acetate_^−1^)	0.83 ± 0.05	0.86 ± 0.08	0.44 ± 0.01	–
	(g_CDW_ g_acetol_^−1^)	–	–	–	0.24 ± 0.09^a^

Growth rate (*µ*), maximum differential biomass-specific substrate uptake rates (*q*_S_^max^) and the apparent biomass substrate yields (*Y*_X/S_*) in respective shaking flask experiments with 1 h and 1.5 h HT PW containing either 5 g yeast extract (YE) L^−1^ or 1 mM reduced glutathione (GSH) as well as 5 g acetol L^−1^ with 5 g YE L^−1^. Error bars depict the SD of ≥ 3 independent experiments

^a^Calculated for the second growth phase (cf. Fig. 2d)

Since YE is an undefined, complex, and expensive supplement, investigations of possible substitutions were conducted that generated targets for strain engineering. We found that a substitution of YE is partially feasible by a supply of 1 mM reduced glutathione (GSH, γ-l-glutamyl-l-cysteinyl-glycine) (cf. Fig. 2b). This low molecular weight thiol plays a primary role for example in detoxification during an abundance of cellular stresses, redox buffering, the methylglyoxal metabolism or directly reacts with lignocellulose-derived inhibitors [56, 57]. The addition of 1 mM GSH to the minimal medium with rel. 3.5% (v/v) 1 h HT PW led to a growth rate of 0.18 ± 0.02 h^−1^ and a net generated biomass of 1.09 ± 0.08 g L^−1^, which are 58 and 46% reduced to that in medium containing 5 g YE L^−1^, respectively. Still, the *q*_S_^max^ of 9.35 ± 0.75 mmol acetate per g CDW per h and 1.13 ± 0.21 mmol acetol per g CDW per h was within the range of the YE supplemented reference (cf. Table 3). Yet, the use of acetol as sole carbon source in the presence of GSH was unsuccessful (data not shown). As *C. glutamicum* naturally produces the alternative low molecular weight thiol mycothiol (MSH, 1D-myo-inositol-2-(*N*-acetyl-l-cysteinyl)amino-2-deoxy-α-d-glucopyranoside) [58], the role of GSH in the respective cultivations remains unclear so far. As already shown for *S. cerevisiae* [59], *Lactococcus lactis* [60] or *C. glutamicum* [61], overproduction of GSH and/or MSH via overexpression of synthesizing pathways might counteract growth inhibiting effects of lignocellulose-derived substrates and thereby increases the overall strain robustness.

In conclusion, we developed a suitable PW pretreatment that allows fast growth of *C. glutamicum* wild-type. Since clarified 1 h HT PW allowed robust growth at a high rate, yielded the highest net generated biomass, and provided adequate acetate and acetol concentrations (cf. Fig. 2b, Table 3, Additional file 1: Table S1), this fraction was used for succeeding experiments. Furthermore, we demonstrated that under aerobic conditions acetol can be used as carbon source, however, only in the presence of YE. Also a substitution of YE by GSH was feasible and generates targets for future strain engineering.

### Pyrolysis water-based 1,2-propanediol production

#### Aerobic 1,2-PDO production with *C. glutamicum* PDO1

**Fig. 3:**
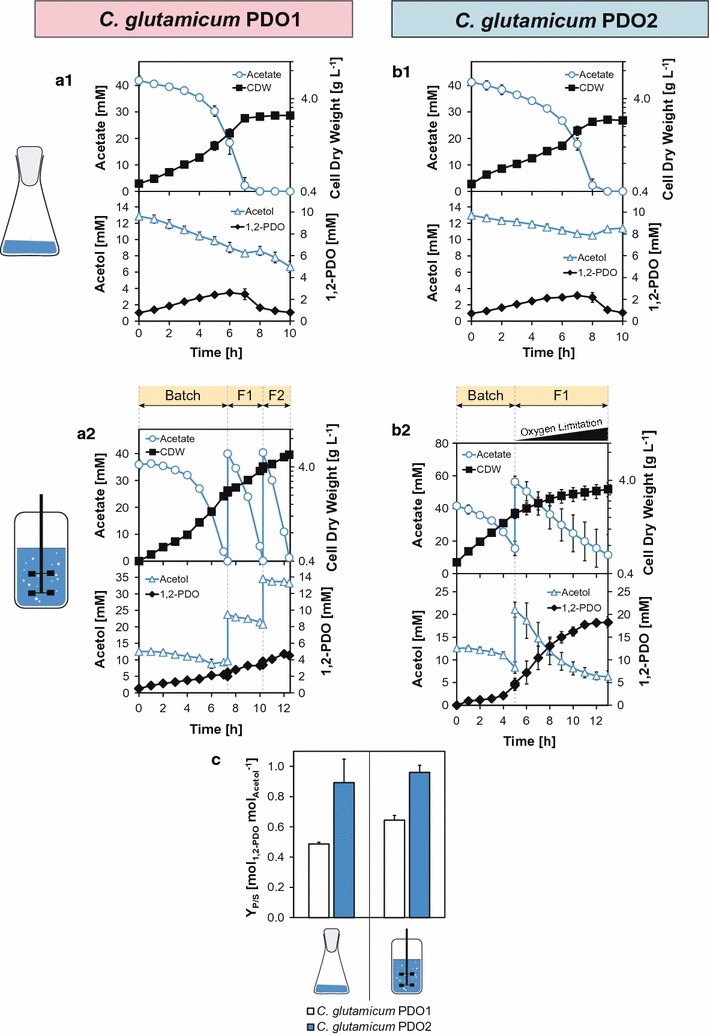
1,2-PDO production using pyrolysis water (PW) with *C. glutamicum* PDO1 (**a**; wild-type + pJUL*gldA*) and *C. glutamicum* PDO2 (**b**; Δ*pqo* Δ*aceE* Δ*ldhA* Δ*mdh* + pJUL*gldA*). Cultivations were performed in shaking flasks (1; CGXII* medium) and bioreactors (2; CGXII** medium) and were supplemented with PW (clarified and exposed to 1 h heat treatment at 80 °C) and yeast extract (YE). Depicted are the concentrations of cell dry weight (CDW) in g L^−1^ and acetate, acetol, and 1,2-PDO in mM over the process time. PW and YE were fed intermittently after the initial batch phase at indicated time points in the aerobic fermentation (**a2**; F1, F2) and two-phase aerobic/microaerobic processes (**b2**; F1). A gradually increasing oxygen deprivation is indicated by a triangle in the feed phase (F1). The product yields (*Y*_P/S_) are shown in mol 1,2-PDO per mol acetol (**c**). Error bars represent SD of triplicate experiments

We conceived a growth-coupled biotransformation exploiting PW as substrate with acetate as main carbon source for biomass generation and acetol as precursor for 1,2-PDO production. Therefore, we cloned the plasmid pJUL*gldA*, which constitutively expresses the *gldA* gene encoding the *E. coli* glycerol dehydrogenase and constructed *C. glutamicum* PDO1 (wild type + pJUL*gldA*). In shaking flask experiments with 1 h HT PW and 5 g YE L^−1^, *C. glutamicum* PDO1 showed a growth rate of 0.23 ± 0.1 h^−1^, a *q*_S_^max^ of 8.8 ± 0.4 mmol acetate per g CDW per h and 1.2 ± 0.1 mmol acetol per g CDW per h and produced 2.6 ± 0.1 mM 1,2-PDO after 6 h of cultivation. After full consumption of acetate, 1,2-PDO was rapidly metabolized and the acetol concentration in the culture broth re-increased (cf. Fig. 3a1). This was most likely catalyzed by a reverse reaction of the glycerol dehydrogenase [62]. The product yield of 0.49 ± 0.01 mol 1,2-PDO per mol acetol indicated that acetol is also used for biomass formation as shown in experiments with the *C. glutamicum* wild-type (cf. Fig. 2d).

Hereafter, we implemented an aerobic intermittent fed-batch fermentation with *C. glutamicum* PDO1 (cf. Fig. 3a2). After the initial batch phase, 1 h HT PW and YE was fed two times (F1 at 7.3 h, F2 at 10.3 h) into the bioreactor resulting in a final concentration of 8.6% (v/v) PW. As trigger for manual PW feeding, a sharp increase of the online DO signal was used, which indicated the complete consumption of acetate (not shown). A 1,2-PDO metabolization as seen above (cf. Fig. 3a1) after full consumption of acetate was therefore prevented. Increasing PW concentrations came along with a reduction of the growth rate (0.26 ± 0.00, 0.19 ± 0.01, 0.12 ± 0.01 h^−1^) and the *Y*_X/S_* (0.72 ± 0.02, 0.67 ± 0.01, 0.59 ± 0.01 g CDW per g acetate) for the batch, F1 and F2 phase, respectively. This was accompanied by a stepwise reduction of the q_acetate_^max^ (9.08 ± 0.11, 6.40 ± 0.35, 4.48 ± 0.09 mmol acetate per g CDW per h) and q_acetol_^max^ (1.36 ± 0.06, 1.23 ± 0.36, 0.37 ± 0.17 mmol acetol per g CDW per h) as well as the *q*_1,2-PDO_^max^ (0.65 ± 0.20, 0.21 ± 0.04, 0.13 ± 0.01 mmol 1,2-PDO per g CDW per h) in the respective phases. After 13 h of cultivation, a net biomass of 4.5 ± 0.0 g L^−1^ was formed and *C. glutamicum* PDO1 produced 4.7 ± 0.0 mM 1,2-PDO with an overall differential *Y*_P/S_ of 0.64 ± 0.03 mol 1,2-PDO per mol acetol (cf. Fig. 3c). The *q*_acetate_^max^ surpassed the q_acetol_^max^ by a ratio of approximately 6:1. This led to an accumulation of acetol as only 18% of the overall supply was consumed. Nevertheless, the process is advanced compared to the respective shaking flask experiments due to a lower ratio of *q*_acetate_^max^ to *q*_acetol_^max^ with *C. glutamicum* wild-type of 8:1 and PDO1 of 7:1 (cf. Additional file 1: Figure S5E).

#### Aerobic and two-phase aerobic/microaerobic 1,2-PDO production with *C. glutamicum* PDO2

The results of the shaking flask and bioreactor cultivations with *C. glutamicum* PDO1 accented the need to balance acetate and acetol uptake for an efficient use of PW as substrate for 1,2-PDO production. Because the glycerol dehydrogenase uses NADH for reduction of acetol to 1,2-PDO, we intended to enhance the cell’s reducing power via oxygen limitation and conceived a two-phase aerobic/microaerobic production process. In comparison to aerobic conditions, it was shown that the NADH/NAD^+^ ratio drastically increases with decreasing oxygen availability [63, 64]. Since *C. glutamicum* produces the fermentation products lactate, acetate, and succinate under oxygen deprived conditions [65], we used *C. glutamicum* Δ*pqo* Δ*aceE* Δ*ldhA* Δ*mdh* [33], which is severely impaired in the capability to secrete these organic acids. Using this strain, a lack of by-product formation under microaerobic conditions will benefit to the overall production performance. *C. glutamicum* Δ*pqo* Δ*aceE* Δ*ldhA* Δ*mdh* was transformed with the plasmid pJUL*gldA*, yielding *C. glutamicum* PDO2, which was initially tested in shaking flask experiments (cf. Fig. 3b1). Compared to *C. glutamicum* PDO1, the newly constructed strain *C. glutamicum* PDO2 exhibited a slightly reduced growth rate and acetate consumption rate (0.23 ± 0.01 vs. 0.18 ± 0.01 h^−1^, 8.8 ± 0.4 vs. 6.2 ± 0.5 mmol acetate per g CDW per h) and an about 50% lowered q_acetol_^max^ (1.2 ± 0.1 vs. 0.7 ± 0.0 mmol acetol per g CDW per h) (cf. Additional file 1: Figure S5C). Although the maximum 1,2-PDO titer was not increased (2.4 ± 0.1 mM) and the *q*_1,2-PDO_^max^ remained comparable (0.6 ± 0.0 mmol 1,2-PDO per g CDW per h), the *Y*_P/S_ was improved by 80% up to 0.89 ± 0.15 mol 1,2-PDO per mol acetol compared to *C. glutamicum* PDO1 (cf. Additional file 1: Figure S5D, Fig. 3c). Additional shaking flask experiments with *C. glutamicum* PDO1 and PDO2 using potassium acetate as sole carbon source demonstrated that 1,2-PDO cannot be produced from this carbon source under the applied conditions (data not shown). The increased yield thus mirrors a reduced acetol uptake that originates from the inability of *C. glutamicum* PDO2 to channel acetol into the central metabolism. Most likely, this is explained by the lack of the lactate dehydrogenase, which is discussed to be involved in the conversion of acetol towards pyruvate [55]. This effect led to an elevated ratio of the *q*_acetate_^max^ to *q*_acetol_^max^ up to 10:1 (cf. Additional file 1: Figure S5E) and allowed the consumption of only 20% of the provided acetol (48% with *C. glutamicum* PDO1).

To improve NADH availability and thereby enhance acetol utilization with *C. glutamicum* PDO2, we established a two-phase aerobic/microaerobic fermentation process in CGXII** medium with 1 h HT PW with YE. In the process, an aerobic phase was succeeded by a feed phase (F1 starting at 5 h), where a low and constant aeration rate gradually increased oxygen limitation with a DO approximating 0% and reduced growth and biomass-specific acetate uptake (cf. Fig. 3b2). Under oxygen shortage, no significant production of the fermentation by-products lactate, succinate, or acetate was detected (data not shown), which most likely gave rise to a higher NADH supply for the GldA catalyzed reaction. In this phase, the *q*_S_^max^ were 3.6 ± 0.9 mmol acetate per g CDW per h and 2.0 ± 0.3 mmol acetol per g CDW per h, respectively. The acetate and acetol uptake rates accordingly converged to a ratio of 2:1 (cf. Additional file 1: Figure S5E), which resulted in an improvement of overall acetol consumption. In fact, 75% of total acetol was metabolized. Due to the fact that *C. glutamicum* PDO2 is unable to funnel acetol into the central metabolism, the majority incorporated carbon is converted into 1,2-PDO with a *Y*_P/S_ close to the theoretical maximum of 0.96 ± 0.05 mol 1,2-PDO per mol acetol. *C. glutamicum* PDO2 finally produced 18.3 ± 1.2 mM 1,2-PDO. With respect to the total carbon content of the 1 h HT PW within the process, the *Y*_P/S_ was 0.11 ± 0.03 C-mol 1,2-PDO per C-mol pretreated PW. This bioprocess accomplished the so far highest overall differential volumetric productivity for a microbial 1,2-PDO production with 1.4 ± 0.1 mmol 1,2-PDO L^−1^ h^−1^ in an engineered producer strain considering entire processes including the period of biomass formation and production. The production parameters are summarized in Table 1, with respect to other published microbial production processes and compared in Additional file 1: Figure S5 for the conducted experiments.

## Conclusions

This study describes the access to a novel value chain implementing microbial fermentation into biorefineries, by using the unexploited side stream pyrolysis water derived from pyrolytic liquefaction of lignocellulosic biomass. Pyrolysis water was demonstrated to be a suitable substrate for *C. glutamicum* and applicable for 1,2-PDO production with engineered strains in growth-coupled biotransformations. Thereby, acetate represents the major substrate for biomass formation, whereas acetol is converted to 1,2-PDO by heterologously expressed glycerol dehydrogenase. Both substrates can be fully metabolized by *C. glutamicum* wild-type, which fans out the scope of pyrolysis water-based products in future biorefinery strategies with engineered derivatives.

## Additional file

**Additional file 1.** Additional figures and tables.

## Abbreviations

PW: pyrolysis water
HT: heat treated
YE: yeast extract

## References

[1] Communiqué of the global bioeconomy summit—making bioeconomy work for sustainable development. The Bioeconomy Council, Independent advisory body to the German Federal Government. Berlin; 2015.

[2] Jungmeier G, Van Ree R, De Jong E, Stichnothe H, De Bari I, Jørgensen H, et al. The “biorefinery fact sheet” and its application to wood based biorefining—case studies of IEA bioenergy task 42 “biorefining.”. Presented at The 6th Nordic Wood Biorefinery Conference (NWBC). Helsinki: VTT Technical Research Centre of Finland Ltd; 2015. p. 6.

[3] Rabaçal M, Ferreira AF, Silva CAM, Costa M (2017). Biorefineries—targeting energy, high value products and waste valorisation.

[4] Cherubini F (2010). The biorefinery concept: using biomass instead of oil for producing energy and chemicals. Energy Convers Manag..

[5] Bridgwater AV, Rabaçal M, Ferreira AF, Silva CAM, Costa M (2017). Biomass conversion technologies: fast pyrolysis liquids from biomass: quality and upgrading. Biorefineries.

[6] Meier D (2017). Pyrolysis oil biorefinery.

[7] Dahmen N, Abeln J, Eberhard M, Kolb T, Leibold H, Sauer J (2016). The bioliq process for producing synthetic transportation fuels. Wiley Interdiscip Rev.

[8] Pfitzer C, Dahmen N, Tröger N, Weirich F, Sauer J, Günther A (2016). Fast pyrolysis of wheat straw in the bioliq pilot plant. Energy Fuels.

[9] Oasmaa A, Sundqvist T, Kuoppala E, Garcia-Perez M, Solantausta Y, Lindfors C (2015). Controlling the phase stability of biomass fast pyrolysis bio-oils. Energy Fuels.

[10] Dahmen N, Henrich E, Dinjus E, Weirich F (2012). The bioliq^®^ bioslurry gasification process for the production of biosynfuels, organic chemicals, and energy. Energy Sustain Soc..

[11] Chen D, Cen K, Jing X, Gao J, Li C, Ma Z (2017). An approach for upgrading biomass and pyrolysis product quality using a combination of aqueous phase bio-oil washing and torrefaction pretreatment. Bioresour Technol.

[12] Zeng A-P, Sabra W (2011). Microbial production of diols as platform chemicals: recent progresses. Curr Opin Biotechnol.

[13] Zhou C-HC, Beltramini JN, Fan Y-X, Lu GQM (2008). Chemoselective catalytic conversion of glycerol as a biorenewable source to valuable commodity chemicals. Chem Soc Rev..

[14] Vennestrøm PNR, Osmundsen CM, Christensen CH, Taarning E (2011). Beyond petrochemicals: the renewable chemicals industry. Angew Chemie Int Ed..

[15] Bennett GN, San KY (2001). Microbial formation, biotechnological production and applications of 1,2-propanediol. Appl Microbiol Biotechnol.

[16] Saxena RK, Anand P, Saran S, Isar J, Agarwal L (2010). Microbial production and applications of 1,2-propanediol. Indian J Microbiol..

[17] Cameron DC, Altaras NE, Hoffman ML, Shaw AJ (1998). Metabolic engineering of propanediol pathways. Biotechnol Prog.

[18] Liebl W, Eggeling L, Bott M (2005). *Corynebacterium* taxonomy. Handb *Corynebacterium glutamicum*.

[19] Becker J, Wittmann C (2015). Advanced biotechnology: metabolically engineered cells for the bio-based production of chemicals and fuels, materials, and health-care products. Angew Chemie Int Ed..

[20] Niimi S, Suzuki N, Inui M, Yukawa H (2011). Metabolic engineering of 1,2-propanediol pathways in *Corynebacterium glutamicum*. Appl Microbiol Biotechnol.

[21] Siebert D, Wendisch VF (2015). Metabolic pathway engineering for production of 1,2-propanediol and 1-propanol by *Corynebacterium glutamicum*. Biotechnol Biofuels.

[22] Kalinowski J, Bathe B, Bartels D, Bischoff N, Bott M, Burkovski A (2003). The complete *Corynebacterium glutamicum* ATCC 13032 genome sequence and its impact on the production of l-aspartate-derived amino acids and vitamins. J Biotechnol.

[23] Wendisch VF, Bott M, Kalinowski J, Oldiges M, Wiechert W (2006). Emerging *Corynebacterium glutamicum* systems biology. J Biotechnol.

[24] Sakai S, Tsuchida Y, Nakamoto H, Okino S, Ichihashi O, Kawaguchi H (2007). Effect of lignocellulose-derived inhibitors on growth of and ethanol production by growth-arrested *Corynebacterium glutamicum* R. Appl Environ Microbiol.

[25] Jojima T, Inui M, Yukawa H, Inui M, Yukawa H (2013). Biorefinery applications of *Corynebacterium glutamicum*. *Corynebacterium glutamicum*.

[26] Arnold S, Moss K, Henkel M, Hausmann R (2017). Biotechnological perspectives of pyrolysis oil for a bio-based economy. Trends Biotechnol.

[27] Cameron DC, Cooney CL (1986). A novel fermentation: the production of R(-)-1,2-propanediol and acetol by *Clostridium thermosaccharolyticum*. Nat Biotechnol.

[28] Clomburg JM, Gonzalez R (2011). Metabolic engineering of *Escherichia coli* for the production of 1,2-propanediol from glycerol. Biotechnol Bioeng.

[29] Altaras NE, Cameron DC (2000). Enhanced production of (R)-1,2-propanediol by metabolically engineered *Escherichia coli*. Biotechnol Prog.

[30] Oude Elferink SJ, Krooneman J, Gottschal JC, Spoelstra SF, Faber F, Driehuis F (2001). Anaerobic conversion of lactic acid to acetic acid and 1,2-propanediol by *Lactobacillus buchneri*. Appl Environ Microbiol.

[31] Jung J-Y, Yun HS, Lee J, Oh M-K (2011). Production of 1,2-propanediol from glycerol in *Saccharomyces cerevisiae*. J Microbiol Biotechnol.

[32] Hanahan D (1983). Studies on transformation of *Escherichia coli* with plasmids. J Mol Biol.

[33] Radoš D, Carvalho AL, Wieschalka S, Neves AR, Blombach B, Eikmanns BJ (2015). Engineering *Corynebacterium glutamicum* for the production of 2,3-butanediol. Microb Cell Fact.

[34] Cordes C, Möckel B, Eggeling L, Sahm H (1992). Cloning, organization and functional analysis of *ilvA*, *ilvB* and *ilvC* genes from *Corynebacterium glutamicum*. Gene.

[35] Sambrook J, Russell DW (2001). Molecular cloning: a laboratory manual.

[36] Dower WJ, Miller JF, Ragsdale CW (1988). High efficiency transformation of *E. coli* by high voltage electroporation. Nucleic Acids Res.

[37] Tauch A, Kirchner O, Löffler B, Götker S, Pühler A, Kalinowski J (2002). Efficient electrotransformation of *Corynebacterium diphtheriae* with a mini-replicon derived from the *Corynebacterium glutamicum* plasmid pGA1. Curr Microbiol.

[38] van der Rest ME, Lange C, Molenaar D (1999). A heat shock following electroporation induces highly efficient transformation of *Corynebacterium glutamicum* with xenogeneic plasmid DNA. Appl Microbiol Biotechnol.

[39] Truniger V, Boos W (1994). Mapping and cloning of *gldA*, the structural gene of the *Escherichia coli* glycerol dehydrogenase. J Bacteriol.

[40] Menkel E, Thierbach G, Eggeling L, Sahm H (1989). Influence of increased aspartate availability on lysine formation by a recombinant strain of *Corynebacterium glutamicum* and utilization of fumarate. Appl Environ Microbiol.

[41] Miwa K, Matsui H, Terabe M, Nakamori S, Sano K, Momose H (1984). Cryptic plasmids in glutamic acid-producing bacteria. Agric Biol Chem.

[42] Chang AC, Cohen SN (1978). Construction and characterization of amplifiable multicopy DNA cloning vehicles derived from the P15A cryptic miniplasmid. J Bacteriol.

[43] Rose RE (1988). The nucleotide sequence of pACYC177. Nucleic Acids Res..

[44] Brosius J, Dull TJ, Sleeter DD, Noller HF (1981). Gene organization and primary structure of a ribosomal RNA operon from *Escherichia coli*. J Mol Biol.

[45] Orosz A, Boros I, Venetianer P (1991). Analysis of the complex transcription termination region of the *Escherichia coli rrnB* gene. Eur J Biochem.

[46] Gibson DG, Young L, Chuang R-Y, Venter JC, Hutchison CA, Smith HO (2009). Enzymatic assembly of DNA molecules up to several hundred kilobases. Nat Methods.

[47] Eikmanns BJ, Metzger M, Reinscheid D, Kircher M, Sahm H (1991). Amplification of three threonine biosynthesis genes in *Corynebacterium glutamicum* and its influence on carbon flux in different strains. Appl Microbiol Biotechnol.

[48] Keilhauer C, Eggeling L, Sahm H (1993). Isoleucine synthesis in *Corynebacterium glutamicum*: molecular analysis of the *ilvB*-*ilvN*-*ilvC* operon. J Bacteriol.

[49] Buchholz J, Schwentner A, Brunnenkan B, Gabris C, Grimm S, Gerstmeir R (2013). Platform engineering of *Corynebacterium glutamicum* with reduced pyruvate dehydrogenase complex activity for improved production of l-lysine, l-valine, and 2-ketoisovalerate. Appl Environ Microbiol.

[50] Buchholz J, Graf M, Blombach B, Takors R (2014). Improving the carbon balance of fermentations by total carbon analyses. Biochem Eng J.

[51] Oasmaa A, Elliott DC, Korhonen J (2010). Acidity of biomass fast pyrolysis bio-oils. Energy Fuels.

[52] Diebold J (2000). A review of the chemical and physical mechanisms of the storage stability of fast pyrolysis bio-oils.

[53] Oasmaa A, Peacocke C. Properties and fuel use of biomass-derived fast pyrolysis liquids: a guide. Finland: VTT Publications; 2010. p. 46.

[54] Wendisch VF, de Graaf AA, Sahm H, Eikmanns BJ (2000). Quantitative determination of metabolic fluxes during coutilization of two carbon sources: comparative analyses with *Corynebacterium glutamicum* during growth on acetate and/or glucose. J Bacteriol.

[55] Kalapos MP (1999). Methylglyoxal in living organisms: chemistry, biochemistry, toxicology and biological implications. Toxicol Lett.

[56] Masip L, Veeravalli K, Georgiou G (2006). The many faces of glutathione in bacteria. Antioxid Redox Signal..

[57] Kim D, Hahn J-S (2013). Roles of the Yap1 transcription factor and antioxidants in *Saccharomyces cerevisiae*’s tolerance to furfural and 5-hydroxymethylfurfural, which function as thiol-reactive electrophiles generating oxidative stress. Appl Environ Microbiol.

[58] Liu Y-B, Long M-X, Yin Y-J, Si M-R, Zhang L, Lu Z-Q (2013). Physiological roles of mycothiol in detoxification and tolerance to multiple poisonous chemicals in *Corynebacterium glutamicum*. Arch Microbiol.

[59] Ask M, Mapelli V, Höck H, Olsson L, Bettiga M (2013). Engineering glutathione biosynthesis of *Saccharomyces cerevisiae* increases robustness to inhibitors in pretreated lignocellulosic materials. Microb Cell Fact.

[60] Li Y, Hugenholtz J, Sybesma W, Abee T, Molenaar D (2004). Using *Lactococcus lactis for* glutathione overproduction. Appl Microbiol Biotechnol.

[61] Liu Y-B, Chen C, Chaudhry MT, Si M-R, Zhang L, Wang Y (2014). Enhancing *Corynebacterium glutamicum* robustness by over-expressing a gene, *mshA*, for mycothiol glycosyltransferase. Biotechnol Lett.

[62] Tang CT, Ruch FE, Lin CC (1979). Purification and properties of a nicotinamide adenine dinucleotide-linked dehydrogenase that serves an *Escherichia coli* mutant for glycerol catabolism. J Bacteriol.

[63] Molenaar D, van der Rest ME, Drysch A, Yücel R (2000). Functions of the membrane-associated and cytoplasmic malate dehydrogenases in the citric acid cycle of *Corynebacterium glutamicum*. J Bacteriol.

[64] Inui M, Murakami S, Okino S, Kawaguchi H, Vertès AA, Yukawa H (2004). Metabolic analysis of *Corynebacterium glutamicum* during lactate and succinate productions under oxygen deprivation conditions. J Mol Microbiol Biotechnol.

[65] Dominguez H, Nezondet C, Lindley ND, Cocaign M (1993). Modified carbon flux during oxygen limited growth of *Corynebacterium glutamicum* and the consequences for amino acid overproduction. Biotechnol Lett.

