# Supplementary material for: Valorization of pyrolysis water: a biorefinery side stream, for 1,2-propanediol production with engineered Corynebacterium glutamicum
Source: Biotechnol Biofuels. 2017 Nov 21;10:277. doi: 10.1186/s13068-017-0969-8 (PMC5697356; doi:10.1186/s13068-017-0969-8)
Supplement: Supplementary file 1 — Additional file 1. Additional figures and tables. [file 13068_2017_969_MOESM1_ESM.docx]

**Additional file 1**

The following information is provided to the article

**Valorization of Pyrolysis Water – a Biorefinery Side Stream – for 1,2-Propanediol Production with engineered *Corynebacterium glutamicum***

in Biotechnology for Biofuels

Julian Lange^a^

Felix Müller^a^

Kerstin Bernecker^a^

Nicolaus Dahmen^b^

Ralf Takors^a^

Bastian Blombach^a^^[[1]](#footnote-1)^

^a^Institute of Biochemical Engineering, University of Stuttgart, D-70569 Stuttgart, Germany

^b^Institute for Catalysis Research and Technology, Karlsruhe Institute of Technology (KIT), D-76344 Eggenstein-Leopoldshafen, Germany

# Additional Figures

## Figure S1


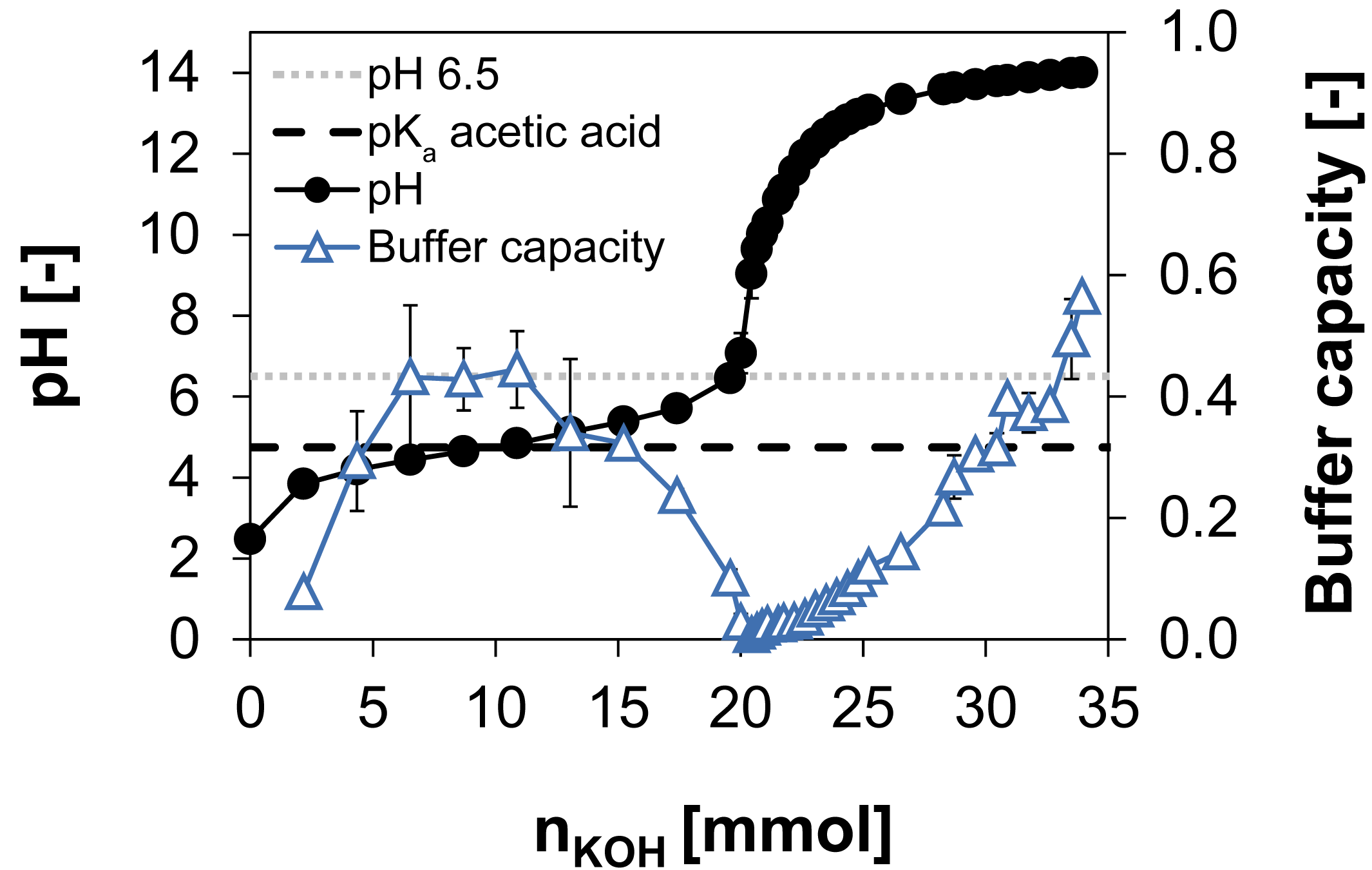


Fig. S1. Titration curve (black circles) of crude pyrolysis water by the addition of potassium hydroxide (from a 4.4 M KOH stock solution). The buffer capacity β (open triangles) was calculated by the differential concentrations of added KOH and the pH change (β = Δc_KOH_ / ΔpH). Horizontal lines delineate the pK_a_ of acetic acid (4.76, dashed line, [1]) and pH 6.5 (dotted line), which was the set point in the pyrolysis water pretreatment and in shaking flask cultivations. Error bars show SD of three independent experiments.

## Figure S2


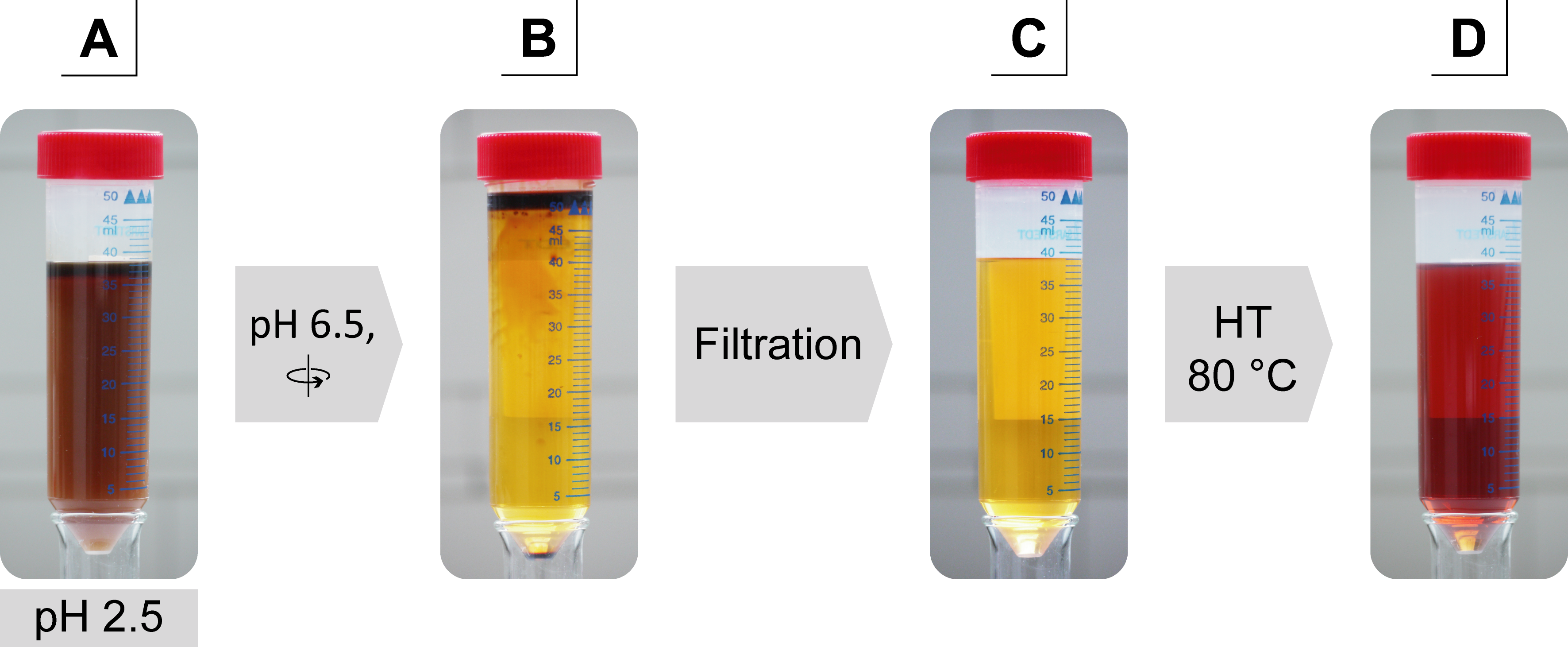


Fig. S2. Sequence of pyrolysis water (PW) pretreatments from left to right: A. Crude PW as stable emulsion of hydrophobic entities and suspended char at pH 2.5. B. Hydrophobic, aqueous and solid phase separation after adjustment of the pH to 6.5 by addition of potassium hydroxide and subsequent centrifugation at 4500 rcf for 30 min. C. Clarified PW after hydrophobic top layer removal and filtration. D. Heat treated (HT) PW (80 °C in open beakers for 1 h). Volume changes are not representative.

## Figure S3


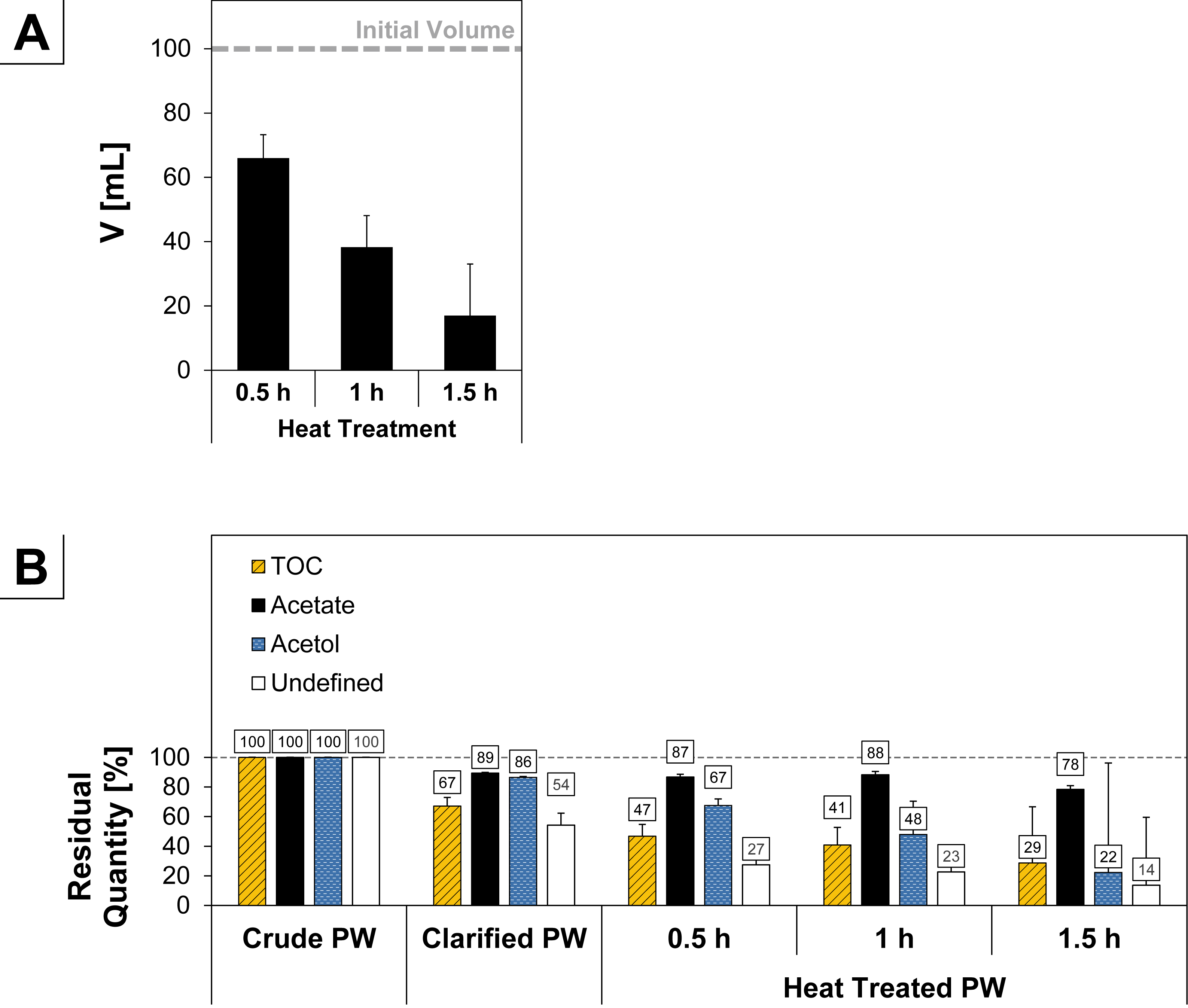


Fig S3. Volume reduction, residual quantities of TOC, acetate and acetol in the course of the applied pyrolysis water (PW) pretreatments. A. Volume reduction of separate PW batches during heat treatment at 80 °C in open beakers. Depicted is the final volume after the respective time of exposure. The initial volume of 100 mL is indicated (dashed line). B. Residual quantities after the treatment are given in relative percent considering the volume loss and are based on total organic carbon (TOC) content, acetate, acetol and undefined entities in g carbon L^-1^ (C-g L^-1^). Error bars represent SD of ≥ 4 independent treatments and measurements.

## Figure S4


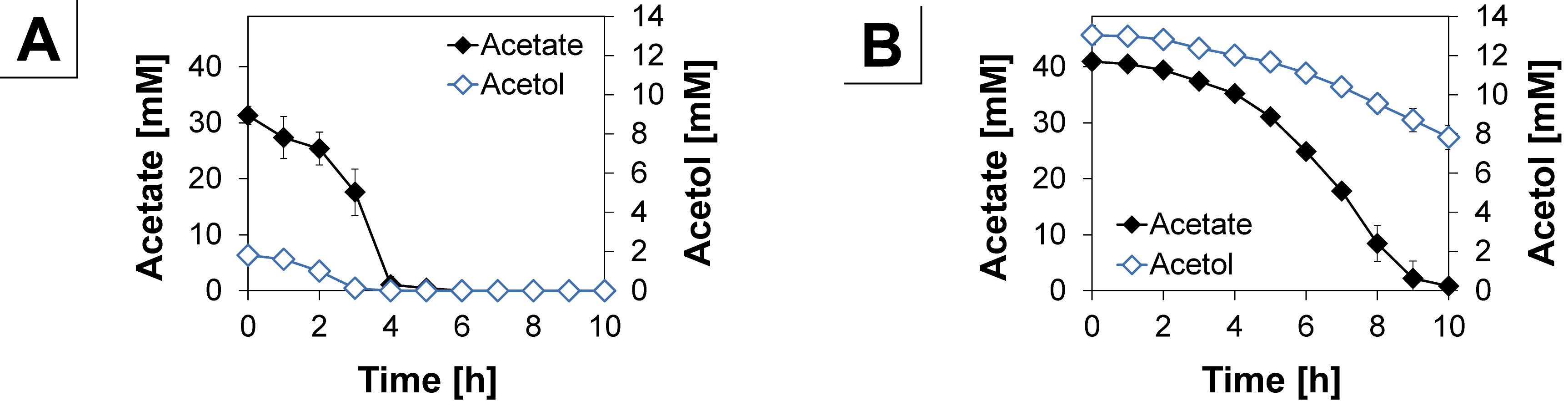


Fig. S4. Consumption of acetate and acetol in the shaking flask experiments shown in Fig. 2 B of *C. glutamicum* wild type in CGXII* medium supplemented with 1.5 h heat treated (HT) pyrolysis water (PW) and 5 g yeast extract L^-1^ (A) and 1 h HT PW supplemented with 1 mM reduced glutathione (B). Error bars represent the SD of ≥ 3 independent experiments.

## Figure S5


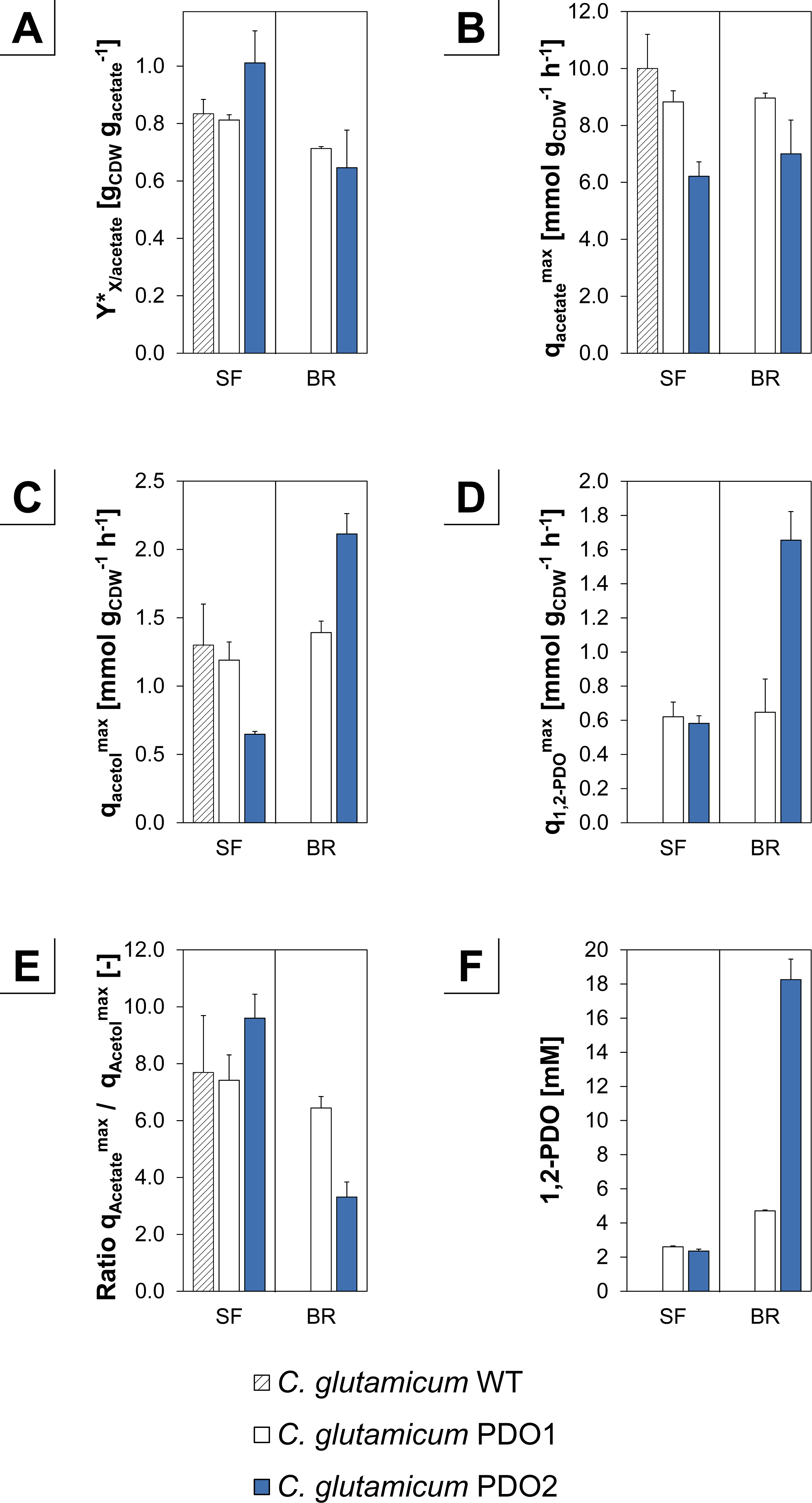


Fig. S5. Comparison of secondary parameters during cultivations of *C. glutamicum* wild type (shaded), *C. glutamicum* PDO1 (white; wild type + pJUL*gldA*) and *C. glutamicum* PDO2 (blue; Δ*pqo* Δ*aceE* Δ*ldhA* Δ*mdh* + pJUL*gldA*) in shaking flasks (SF; CGXII* medium) and bioreactors (BR; CGXII** medium). Media were supplemented with 1 h HT PW (pyrolysis water clarified and exposed to 1 h heat treatment at 80 °C) and 5 g yeast extract (YE) L^-1^. A. Apparent biomass yield (Y_X/S_*) in g cell dry weight (CDW) per g acetate. For bioreactor cultivations the yield is given as an overall differential value. B-D. Biomass specific rates were calculated differentially within a 1 h timeframe and maximum values given over the period of the entire process: maximum biomass specific acetate uptake rate (B; q_Acetate_^max^), maximum biomass specific acetol uptake rate (C; q_Acetol_^max^), and maximum biomass specific 1,2-PDO production rate (D; q_1,2-PDO_^max^). E. Ratio of the respective maximum biomass specific uptake rates of acetate and acetol. F. Maximum titers of 1,2-PDO throughout the cultivation. Error bars represent standard deviations of triplicate experiments.

# Additional Tables

## Table S1

Tab. S1. True concentrations of the total organic carbon (TOC), acetate and acetol as either g carbon L^-1^ (C-g L^-1^) or mol L^-1^ (M) of different pretreatments of pyrolysis water (PW). Treatment from top to bottom: crude PW; clarified PW (after adjustment from pH ~ 2.5 to 6.5 with 10 mM KOH; solid and hydrophobic phase removal); systematically heat treated (HT) PW at 80 °C for 0.5 h, 1 h, and 1.5 h in open vessels. The undefined amount of organic carbon was determined by the subtraction of the acetate and acetol concentrations from the TOC. The volume loss during heat treatment is not considered here. SD of ≥ 4 independent treatments and measurements. Error for the undefined fraction given relatively to the TOC analysis SD.

| **Pretreatment** | |  | **TOC** | | |  | **Acetate** | | | | | |  | **Acetol** | | | | | |  | **Undefined** | | |
| --- | --- | --- | --- | --- | --- | --- | --- | --- | --- | --- | --- | --- | --- | --- | --- | --- | --- | --- | --- | --- | --- | --- | --- |
|  |  |  | **[C-g L^-1^]** | | |  | **[C-g L^-1^]** | | | **[M]** | | |  | **[C-g L^-1^]** | | | **[M]** | | |  | **[C-g L^-1^]** | | |
| **Crude PW** | |  | 106 | ± | 11 |  | 22 | ± | 1 | 0.93 | ± | 0.02 |  | 18 | ± | 1 | 0.50 | ± | 0.02 |  | 66 | ± | 7 |
| **Clarified PW** | |  | 71 | ± | 13 |  | 20 | ± | 1 | 0.83 | ± | 0.04 |  | 16 | ± | 1 | 0.43 | ± | 0.02 |  | 36 | ± | 6 |
| **HT PW** | **0.5 h** |  | 80 | ± | 7 |  | 30 | ± | 2 | 1.23 | ± | 0.08 |  | 18 | ± | 1 | 0.50 | ± | 0.02 |  | 32 | ± | 3 |
|  | **1 h** |  | 131 | ± | 27 |  | 44 | ± | 11 | 1.84 | ± | 0.45 |  | 20 | ± | 8 | 0.55 | ± | 0.22 |  | 67 | ± | 14 |
|  | **1.5 h** |  | 223 | ± | 91 |  | 155 | ± | 70 | 6.45 | ± | 2.93 |  | 24 | ± | 1 | 0.65 | ± | 0.02 |  | 45 | ± | 18 |

# References

1. Serjeant EP, Dempsey B. Ionisation constants of organic acids in aqueous solution. Oxford, New York: Pergamon Press; 1979.

1. Corresponding author: Dr. Bastian Blombach (blombach@ibvt.uni-stuttgart.de), Institute of Biochemical Engineering, University of Stuttgart, Allmandring 31, D-70569 Stuttgart, Germany. [↑](#footnote-ref-1)
